# pH/hypoxia programmable triggered cancer photo-chemotherapy based on a semiconducting polymer dot hybridized mesoporous silica framework†
†Electronic supplementary information (ESI) available. See DOI: 10.1039/c8sc02408a


**DOI:** 10.1039/c8sc02408a

**Published:** 2018-07-27

**Authors:** Da Zhang, Zhixiong Cai, Naishun Liao, Shanyou Lan, Ming Wu, Haiyan Sun, Zuwu Wei, Juan Li, Xiaolong Liu

**Affiliations:** a MOE Key Laboratory for Analytical Science of Food Safety and Biology , State Key Laboratory of Photocatalysis on Energy and Environment , College of Chemistry , Fuzhou University , Fuzhou 350116 , P. R. China . Email: lijuan@fzu.edu.cn; b The United Innovation of Mengchao Hepatobiliary Technology Key Laboratory of Fujian Province , Mengchao Hepatobiliary Hospital of Fujian Medical University , Fuzhou 350025 , P. R. China . Email: xiaoloong.liu@gmail.com; c Key Laboratory of Biomedical Information Engineering of Ministry of Education , Institute of Biomedical Analytical Technology and Instrumentation , School of Life Science and Technology , Xi’an Jiaotong University , Xi’an 710049 , P. R. China; d Department of Anesthesiology , Beijing Anzhen Hospital , Capital Medical University , Beijing 100029 , P. R. China

## Abstract

Although photothermal therapy (PTT) has become a compelling strategy for cancer therapy, few studies concern the physiological consequences of PTT ablation. Herein, we discover that PTT-induced hyperthermia can aggravate tumor hypoxia, which may increase the risk of tumor recurrence and reduce PTT efficacy. We thus integrated the pH/hypoxia-triggered Fe(iii)-banoxantrone (AQ4N) prodrug and semiconducting polymer dots (SPs) for programmable triggered cancer photothermal-chemotherapy. A SP-hybridized mesoporous silica framework, decorated by dopamine and polyethylene glycol, named PPMSF, was synthesized by a simple method, and then served as an efficient photo-absorbing agent (PTA) and drug carrier. Fe(iii)-AQ4N and Mn(ii) were then coordinated with PPMSF (abbreviated Mn-APPMSF) *via* coordination effects. The nanohybrids exhibited tumor micro-environment pH triggered drug release. Under the irradiation of NIR light, magnetic resonance imaging (MRI) tracked the accumulation of the nanohybrids in tumors which then destroyed tumor cells by local hyperthermia, this can consequently aggravate the tumor hypoxia levels. Intriguingly, the aggravated hypoxia can further enhance the reduction of AQ4N to significantly improve therapeutic efficacy and effectively inhibit tumor growth when compared with traditional PTT. These results indicate the potential of our nanohybrids as a programmable synergistic agent for cancer therapy.

## Abstract 2

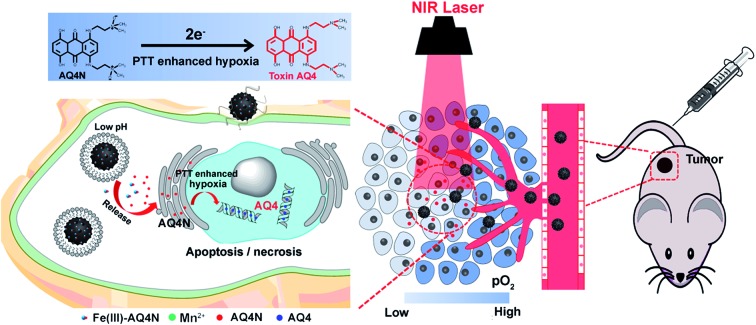
We have integrated the pH/hypoxia-triggered Fe(iii)-banoxantrone (AQ4N) prodrug and semiconducting polymer dots (SPs) for programmable triggered cancer photothermal-chemotherapy.

## Introduction

Cancer is now recognized as a systematic disease rather than a solo abnormality. Different types of benign cells surround the milieu of a tumor and actively facilitate malignant progression in a three-dimensional structure.^1^ It is an increasingly recognized fact that tumor micro-environments (TMEs) can induce abnormal tissue functions and play an essential role in tumor growth, recurrence and metastasis.^2^ The heterogeneity of the TME can instill a number of biological barriers for cancer therapy. Hypoxia and acidic pH are important contributors to the heterogeneity of the TME.^3^ The development of hypoxia and the acidic conditions of tumors can produce substantial cellular stress that drives adaptation mechanisms, *e.g.* altered metabolism, the epithelial–mesenchymal transition, pH regulation and angiogenesis.^4^ In addition, the hypoxia and low pH conditions of TMEs can diminish the immune response of tumor cells and cause resistance to chemotherapy, radiotherapy and photodynamic therapy (PDT).^3^–^5^ With advances in the nanotechnology of bio-medicine, various nanotherapeutic agents have been investigated to enhance cancer therapy such as targeted chemotherapy, photo-therapy (PTT or PDT) and immunotherapy.^6^–^9^ Nevertheless, the antitumor efficacy of these single therapeutic strategies is not satisfactory. Therefore, a combination of dual/multi-model therapeutic strategies could be an effective approach for improving therapeutic efficacy.^10^–^12^

Photothermal therapy (PTT) is an oxygen-independent strategy to ablate malignant carcinomas with the assistance of photo-absorbing agents (PTAs) under near-infrared (NIR) laser irradiation.^13^,^14^ On exposure to a NIR laser at an appropriate wavelength, PTAs can convert electromagnetic wave energy to supply local hyperthermia in the range from 41 °C to 47 °C, or even higher, to destroy tumors. However, the extremely high temperature elevation will cause tissue destruction with denatured proteins, and may additionally damage surrounding healthy tissue and hamper the host immune response.^15^ On the other hand, insufficient hyperthermia induced by different temperature spatial distributions of tumors during PTT may lead to ineffective tumor treatment.^16^ Importantly, in our recent research we found that hyperthermia (*T* > 46 °C) induced by PTT aggravated the hypoxia level in TMEs, resulting in overexpression of hypoxia inducible factor-1 (HIF-1). Additionally, banoxantrone (AQ4N) is a novel bio-reductive prodrug that shows minimum toxicity in oxic cells, but strong antitumor activity in hypoxic cells by an enzymatic process *via* bio-reduction to its metabolite AQ4.^17^ Our previous work has demonstrated that enhancement of the local hypoxia level of tumors could significantly improve the reduction of AQ4N to toxic AQ4 to improve chemo-therapeutic efficacy.^10^,^11^ Therefore, a combination of PTT and the PTT-aggravated hypoxia activated AQ4N prodrug might be an effective strategy to enhance the cancer therapy of traditional PTT in preclinical models.

**Fig. 1:**
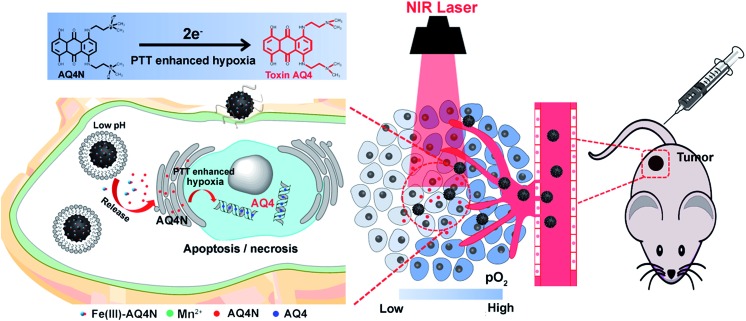
Schematic illustration of the functional principle of Mn-APPMSF in tumor microenvironments. In cancerous cells, AQ4N prodrugs were released from Mn-APPMSF by acidic environment induced cleavage of the coordination bonds between Fe(iii) and the prodrug. The PTT induced hyperthermia could aggravate the tumor hypoxic microenvironment, which could further enhance the bio-reduction of AQ4N to toxic AQ4 for synergistic cancer therapy.

In this study, we integrated semiconducting polymer dots (SPs) and pH/hypoxia-sensitive Fe(iii)-AQ4N prodrugs to form programmed triggered nanohybrids for enhanced cancer photo-chemotherapy (Fig. 1). In the nanohybrids, a dopamine (DA) and polyethylene glycol (PEG) decorated SP-hybridized mesoporous silica framework (PPMSF) was used as an efficient PTA and drug nanocarrier. Fe(iii)-AQ4N acted as an acidic/hypoxia dual-sensitive coordination drug. Mn(ii) ions, chelated on PPMSF, were used as an MRI contrast agent. Thus, the inherent porous characteristics of PPMSF could be used as a template to load Fe(iii)-AQ4N prodrugs and chelate with Mn(ii) ions through coordination effects. Related to the enhanced permeability and retention (EPR) effects, the effective accumulation of nanohybrids in solid tumors was monitored by MRI, and the AQ4N prodrug was released and penetrated into the hypoxic region of solid tumors by acidic-cleavage of the coordination bonds between AQ4N and Fe(iii). Upon 670 nm laser irradiation, these nanohybrids could produce local hyperthermia to destroy tumor cells, and consequently aggravate tumor hypoxia that could improve the bio-reduction of AQ4N for enhanced cancer photo-chemotherapy.

## Results and discussion

### Fabrication and characterization of dopamine inspired PMSF (PPMSF)

**Fig. 2:**
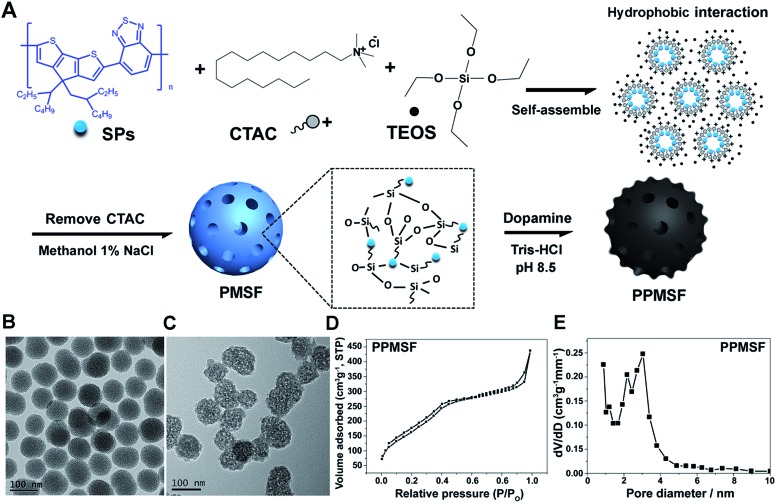
(A) Schematic view of the prepared PPMSF; representative TEM images of PMSF (B) and PPMSF (C); (D) the N_2_ sorption isotherm and (E) the DFT pore size distribution for PPMSF.

The synthesis procedure for PPMSF hybrid nanoparticles is illustrated in Fig. 2A. Briefly, PCPDTBT dots (SPs) were prepared by nanoprecipitation according to previously reported works.^18^–^23^ Afterwards, the hydrophobic SPs were encapsulated into the inner core of cetyltrimethyl ammonium chloride (CTAC, as a template) by a self-assembly method in an alkaline environment for 1 h through hydrophobic interactions, subsequently tetraethylorthosilicate (TEOS, as a silicon source) was gently added to the above mixture at 80 °C for condensation around the CTAC template. After cooling down, the resultant materials were re-suspended in methanol (containing 1% NaCl) to remove the CTAC.^24^ Finally, the PMSF nanoparticles were obtained. The size and morphology of PMSF nanoparticles were investigated by TEM (Fig. 2B), which exhibited a very uniform size distribution around 90 ± 5.7 nm with regular spherical morphology and a clear porous structure. Of note, it was difficult to directly observe SPs inside PMSF nanoparticles due to the low contrast of organic nanoparticles.^25^ However, the color of PMSF changed from ivory to wathet-blue when SPs were doped (Fig. S1†). The existence of SPs inside PMSF nanoparticles was further confirmed by EDS analysis. As shown in Fig. S2,† PMSF nanoparticles consisted of S derived from SPs, and O/Si derived from TEOS, indicating the existence of SPs inside PMSF. The pore size of PMSF was evaluated by N_2_ adsorption–desorption isotherm experiments.^26^ The average pore diameter of PMSF, calculated by the Barrett–Joyner–Halenda (BJH) model from the adsorption branch of isotherms, was *ca.* 3.04 nm with a relatively narrow pore size distribution. The Brunauer–Emmett–Teller (BET) surface area and BJH pore volume were also calculated, *i.e.*, 613.6 m^2^ g^–1^ and 0.698 cm^3^ g^–1^, respectively, which could guarantee high drug loading efficiency (Fig. S3†).

**Fig. 3:**
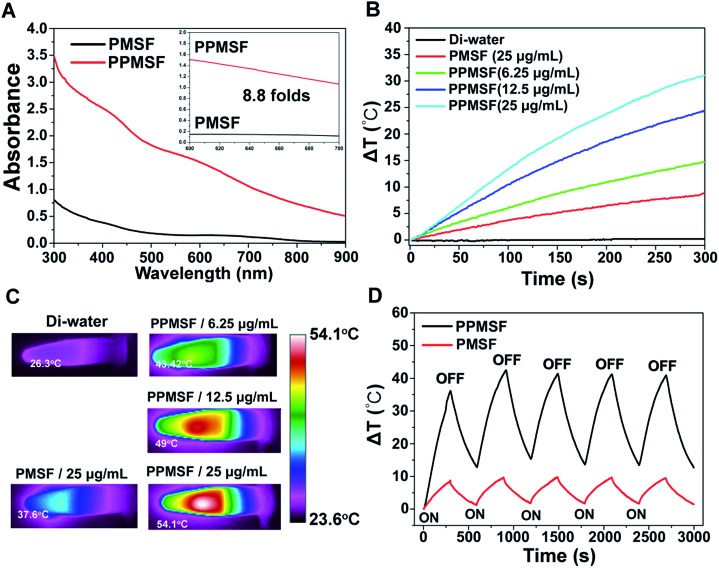
(A) UV-vis-NIR spectra of PMSF and PPMSF; (B) temperature increases and (C) IR thermal images of PMSF and PPMSF; (D) temperature increase curves of PMSF and PPMSF undergoing five rounds of laser irradiation with on/off cycling (670 nm, 1 W cm^–2^).

SPs with excellent photothermal conversion ability have been used as a new class of PTA for PTT.^18^–^23^ However, there have been few studies on the design and preparation of SP nanoparticles (SNPs) for improving PTT effects based on structure/component–property relationships.^18^,^27^ Some published literature has demonstrated that dopamine (DA)-adsorbed nanoparticles could improve PTT effects by enhanced NIR absorbance, one of the key factors to determine the photothermal conversion ability (PCA) of PTAs.^28^–^31^ Thus, the PMSF nanoparticles were incubated with DA for only 2 h under oxidative conditions (pH 8.5) to form DA-adsorbed nanoparticles (named PPMSF). As shown in the TEM image (Fig. 2C), the diameter of PPMSF was 93 ± 5.9 nm on average with regular spherical morphology and a clear porous structure. The color of PMSF changed from wathet-blue to dark grey after DA adsorption (Fig. S1†). The pore size of PPMSF was further evaluated by the “N_2_ adsorption–desorption isotherm” experiment. As shown in Fig. 2D and E, PPMSF nanoparticles showed a porous structure with pore size around 2.75 nm. DLS results suggested that DA was successfully adsorbed on PMSF, as the hydrodynamic size increased from 174 nm to 212 nm (Fig. S4†). Moreover, UV-vis spectra were then analyzed to confirm the enhancement of the absorption intensity of PPMSF. As shown in Fig. 3A, the absorbance of PPMSF was much higher (about 8.8 fold, calculated at 670 nm) than that of unmodified PMSF due to the additional absorbance of DA in the range 350 to 900 nm. The PCA of PPMSF was further investigated with laser irradiation (670 nm, 1 W cm^–2^, 5 min). As shown in Fig. 3B, the temperature of PPMSF dispersions in PBS buffer containing 6.25, 12.5 and 25 μg mL^–1^ of SPs increased by 14.7 °C, 24.4 °C and 31.1 °C, respectively. For the 25 μg mL^–1^ concentration, the temperature of PPMSF increased up to 59.1 °C after irradiation, which was significant higher than the 38.4 °C of PMSF. The temperature elevation by PPMSF (31.1 °C) was about 3.5 fold higher than that by PMSF (8.8 °C). While, the DI-water temperature was only scarcely increased (0.2 °C) due to the negligible absorption of water in this region under laser irradiation and these results were further confirmed by IR thermal images (Fig. 3C). To further compare the photothermal conversion ability of PMSF and PPMSF, the photothermal conversion efficiency (*η*) was measured according to the energy balance of the system.^32^ As shown in Fig. S5,† the energy output was determined by switching off the laser after reaching the steady state of the PMSF and PPMSF solutions, and then the temperature decay curves were measured. The photothermal conversion efficiency (*η*) of PPMSF was determined to be 54.19%, which was about 1.8-fold higher than that of PMSF (28.77%). Additionally, the photothermal stability of PPMSF was investigated since it is one of the most important factors in PTT under the irradiation of NIR laser. PPMSF and PMSF were then subjected to five repeated irradiations with 10 min of laser on/off cycling. As shown in Fig. 3D, both PPMSF and PMSF maintained satisfactory photostability during repeated irradiation, and without undergoing any loss of photothermal conversion ability. Furthermore, compared with PMSF, rapid temperature rising could be observed in PPMSF which was attributed to the enhanced absorbance by DA.^27^–^30^

### Fabrication and characterization of Mn-APPMSF

**Fig. 4:**
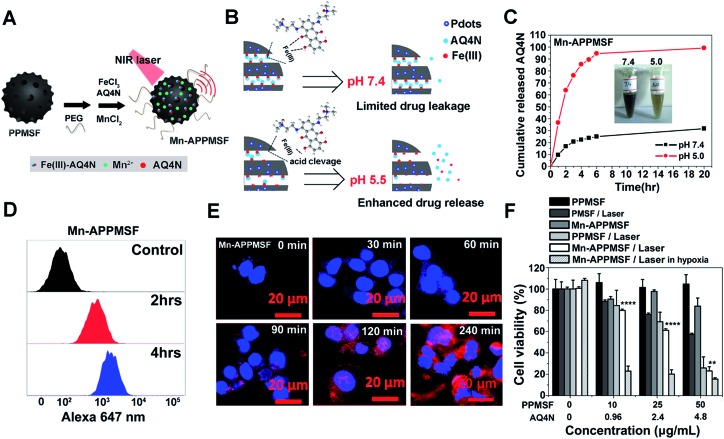
(A) Schematic view of the prepared PPMSF; (B) and (C) the cumulative AQ4N release kinetics from Mn-APPMSF in different pH conditions; (D) flow cytometry analysis of AQ4N inside HepG2 cancer cells after incubation with Mn-APPMSF for 2 and 4 h, respectively. (E) Confocal results of HepG2 cells treated with Mn-APPMSF for 0.5, 1, 1.5, 2 and 4 h. Scale bar, 20 μm. (F) Cell viability of HepG2 cells treated with PMSF, PPMSF, and Mn-APPMSF with/without laser irradiation (670 nm, 1 W cm^–2^) in aerobic or hypoxic conditions (*n* = 5), respectively. The statistical analysis was performed using a two-tailed paired Student’s *t*-test (**p < 0.01, ****p < 0.0001).

It was found in our previous research that hyperthermia (*T* > 46 °C) induced by PTT could aggravate the hypoxia level in TMEs, which may lead to ineffective tumor treatment (unpublished). We thus attempted to combine the hypoxia-activated AQ4N prodrug and the PTA of PPMSF to subdue the above shortfall of PTT for enhanced cancer therapy. To improve the water stability of PPMSF, PEG–(NH_2_)_2_ was conjugated onto the surface of PPMSF *via* Michael addition and/or Schiff base reactions. Moreover, to further avoid non-specific drug release at physiological conditions, the “host–metal–guest” structure of PPMSF-Fe(iii)-AQ4N (named APPMSF) was formed by the immobilization of AQ4N onto PPMSF through Fe(iii)-mediated coordination effects, which might smartly respond to tumor environments such as low pH conditions (Fig. 4A and B). To further track the nanoparticles and monitor tumor locations, APPMSF was then chelated with Mn(ii) ions *via* phenolic hydroxyl coordination effects. The results from the EDS spectrum clearly proved the existence of O, S, Si, Mn and Fe in Mn-APPMSF (Fig. S6†). The amounts of Fe(iii) and Mn(ii) in Mn-APPMSF (0.1 mg mL^–1^) determined from ICP-MS data were approximately 9.6 μg mL^–1^ and 1.12 μg mL^–1^. TEM images suggested Mn-APPMSF was well dispersed in PBS (pH 7.4) with the diameter of 112.7 ± 8.3 nm on average (Fig. S7†). UV-vis spectra were then analyzed to confirm the successful loading of Fe(iii)-AQ4N onto PPMSF. As shown in Fig. S8,† the characteristic absorption peaks of AQ4N ranging from 500 nm to 700 nm were clearly observed in Mn-APPMSF (red line), and these peaks correspond to the characteristic peaks of AQ4N,^11^,^17^ indicating the successful loading of AQ4N onto PPMSF through Fe(iii)-mediated coordination effects with a loading efficiency about 8.27%.

### pH responsive release of AQ4N and cellular uptake of Mn-APPMSF

According to previous reports,^10^,^11^,^33^–^35^ coordination polymers could be degraded in acidic conditions since protons compete with the metal ions to bind with the ligands. The pH responsive release profile of AQ4N in Mn-APPMSF was investigated under various pHs (7.4 and 5.0). The amount of AQ4N released from Mn-APPMSF was quantified by detecting the AQ4N absorbance in the supernatant at various times. As shown in Fig. 4B and C, the released percentage of AQ4N from Mn-APPMSF after 20 h incubation was only 31.6% at pH 7.4. In contrast, the released percentage of AQ4N at pH 5.0 was dramatically increased up to 94.5% even at the initial 6 h time point, indicating that our Mn-APPMSF could smartly respond to acidic conditions including the tumor microenvironment for responsive drug release. Since sufficient internalization of nanoparticles by tumor cells is crucial for cancer therapy,^20^–^27^ we thus investigated cellular internalization of Mn-APPMSF in HepG2 cells using FACS to study the internalization efficiency quantitatively. As shown in Fig. 4D, the AQ4N fluorescence intensity in Mn-APPMSF (excited at 633 nm) was time-dependently increased compared with the control.

Moreover, to further verify whether the fluorescence signals indeed came from loaded AQ4N, a confocal microscopy study was also conducted. As shown in Fig. 4E, AQ4N red fluorescence signals (633 nm laser excitation) could be clearly detected in the cytoplasm of HepG2 cells only after 30 min incubation. With prolonged incubation time, the AQ4N fluorescence intensity from Mn-APPMSF also significantly increased in the cytoplasm of HepG2 cells in aerobic conditions.

### Synergistic antitumor efficacy of Mn-APPMSF

Since Mn-APPMSF can be efficiently internalized by HepG2 cells, we then analyzed the synergistic photothermal/chemotherapy efficiency of Mn-APPMSF by CCK8 assay. The HepG2 cells were then incubated with PMSF, PPMSF and Mn-APPMSF with/without laser irradiation (670 nm, 1 W cm^–2^) in aerobic or hypoxic conditions, respectively. As shown in Fig. 4F, the PPMSF alone as a drug delivery system (DDS) showed a relatively low cytotoxicity for HepG2 cells without irradiation, and the viable cells remained at more than 90% within 48 h incubation. In contrast, with laser irradiation, the cell viability of PPMSF treated cells significantly decreased to 25.9% compared with the 57.5% of PMSF treated cells, indicating that DA significantly enhanced the PTT effect of PMSF. In the meantime, the cell viability of Mn-APPMSF treated cells decreased to 23.1% in the presence of laser irradiation, which was slightly lower than that of PPMSF treated cells, and this might be because of the slight toxicity of AQ4N in aerobic conditions (Fig. S10†). Nevertheless, the cell viability of Mn-APPMSF treated cells sharply declined to 15.48% in the presence of laser irradiation in hypoxic conditions due to the further enhanced cytotoxicity of AQ4N by hypoxia, suggesting the excellent synergistic anticancer efficacy of our Mn-APPMSF in hypoxic conditions. Furthermore, Annexin V-FITC/PI staining was conducted to analyze the apoptosis and necrosis of HepG2 cells treated with Mn-APPMSF. As shown in Fig. S11,† most of the HepG2 cells in the control conditions (untreated and non-irradiated cells) were localized in the lower left quadrant with a cell viability more than 94.58%. Similarly, most control cells remained alive (92.69% of viable cells) even with NIR laser irradiation (670 nm, 1 W cm^–2^), indicating the negligible phototoxicity of the laser itself. In contrast, the cell viability significantly decreased to 63.3% in PPMSF treated HepG2 cells with laser irradiation, which was significantly lower than in PMSF treated cells (83.08%). More strikingly, once the Mn-APPMSF treated cells were irradiated with external light in hypoxic conditions, the percentage of viable HepG2 cells sharply decreased to 39.68%, which was significantly lower than that of the PPMSF treated cells with irradiation. Such findings clearly prove the remarkably and synergistically increased antitumor effects of our Mn-APPMSF through the combination of photothermal therapy and hypoxia-triggered chemotherapy.

### *In vitro* and *in vivo* MRI investigations

Next, we investigated the serum stability of Mn-APPMSF in PBS buffer (pH 7.4) with 10% FBS to mimic the *in vivo* environment. Meanwhile, the PBS buffer without adding FBS was used as a control. As shown in Fig. S9,† even upon adding 10% FBS, the final released percentage of AQ4N from Mn-APPMSF after 20 h was 34.9%, which was comparable with its release profile in PBS buffer without FBS (30.09%). The influence of FBS on the stability of NPs is still tolerable.

**Fig. 5:**
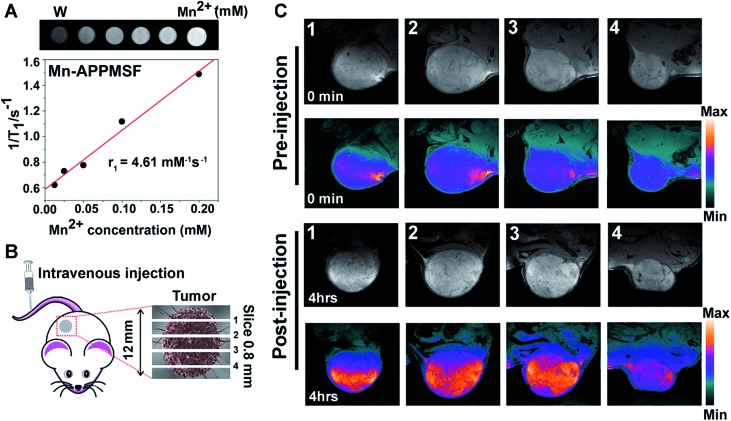
(A) The proton *T*_1_ relaxation rate at different concentrations of Mn(ii) from Mn-APPMSF in a 9.4 T magnetic field. (B) and (C) show representative *T*_1_-weighted MRI images of mice pre- and post-intravenous injection of Mn-APPMSF of different sections from the top to the bottom of the mouse tumor, slice 0.8 mm.

Prior to photo-chemotherapy *in vivo*, detailed information of the tumor including location and size must be identified before treatment to minimize side effects.^36^,^37^ MRI, one of the most powerful strategies for whole body imaging with high spatial resolution and minimal side effects in patients, is also extensively used in tumor diagnosis.^38^*T*_1_ contrast agents of manganese(ii), the most promising clinical contrast agents, not only afforded high *r*_1_ relaxivities for MRI imaging, but also contain a micronutrient which is essential for the function of physiological processes compared with the high toxicity of Gd(iii) based contrast agents.^39^–^41^ Thus, the MRI imaging contrast enhancement ability of our Mn-APPMSF was investigated using a 9.4T MR imaging system (Siemens Magnetom Trio system). As shown in Fig. 5A, a positive MR imaging enhancement of Mn-APPMSF was observed compared with water, and its *T*_1_-weighted MR images became brighter along with the increase in concentration. The *T*_1_-relaxation time for each sample at 20 °C was also recorded, and the results showed that Mn-APPMSF could shorten the *T*_1_-relaxation time. Further investigation of the recorded longitudinal rates showed a linear correlation with Mn-APPMSF concentration (Fig. 5A). The longitudinal coefficient relaxivity value, *r*_1_, calculated from the slope of the plot of 1/*T*_1_*versus* sample concentration, was 4.61 mM^–1^ S^–1^. To further prove the positive signal enhancement ability of MR imaging of Mn-APPMSF *in vivo*, the prepared NPs were intravenously injected into HepG2 tumor bearing mice. After 4 h of injection, the tumors were measured from top to bottom with an interval slice of 0.8 mm. As shown in Fig. 5B and C, the MRI signals were significantly increased after Mn-APPMSF injection compared with the pre-injection section, and showed a very homogenous contrast agent distribution within tumor internal slices of 2 and 3, indicating the good tumor permeation of our nanohybrids.

### Tumor xenografts and *in vivo* pH/hypoxia programmable triggered photothermal/chemotherapy

The *in vivo* biodistribution of our nanocomposite was first investigated by intravenously injecting our Mn-APPMSF into HepG2 tumor bearing mice. After injection for 4 h, the mice were sacrificed and then the amount of Si (which only existed in our nanocomposite) in the tumors and different organs was quantified by ICP-MS (*n* = 3). The results clearly showed that our Mn-APPMSF nanoparticles were mainly accumulated in the liver, tumor, kidney, spleen and lung (Fig. S12A†). In addition, the tumor uptake percentage of Mn-APPMSF after injection of 4 h was about 5.7% ID g^–1^ at 4 h. To further investigate the circulation half-life of our nanocomposites, blood was collected at different time points (0.5, 1, 2, 4, 6, 8, 10, and 24 h) from mice which were intravenously injected with our Mn-APPMSF. Then, the amount of Si from Mn-APPMSF in the blood was determined by ICP-MS, and the pharmacokinetics of our nanocomposites were determined by the content change of Si at different time points. The results are shown in Fig. S12B,† and indicate that our Mn-APPMSF nanoparticles showed prolonged blood circulation up to 24 h, with a circulation half-life of 8.2 h.

**Fig. 6:**
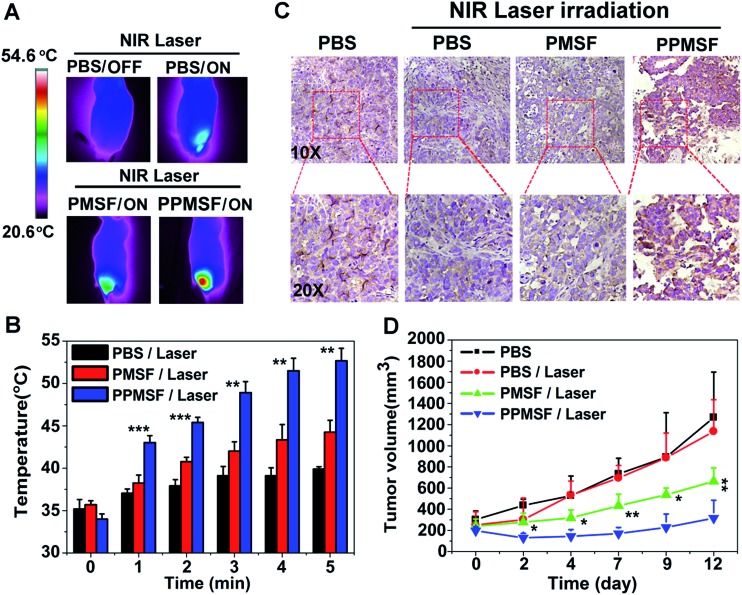
(A) Thermal graphic images of tumor-bearing nude mice after intravenous injection of PBS, PMSF, and PPMSF for 4 h, which were subsequently exposed to laser irradiation (670 nm, 1 W cm^–2^) for 5 min; (B) temperature change at the tumor sites with laser irradiation (670 nm, 1 W cm^–2^) under the indicated conditions (*n* = 4). The statistical analysis was performed using a two-tailed paired Student’s *t*-test (**p < 0.01, ***p < 0.001). (C) *Ex vivo* immunohistochemical staining (HIF-1α) of tumor slices obtained from differently treated mice with or without laser irradiation (670 nm) after 24 h of injection. (D) Tumor volume change of tumor bearing mice after indicated treatments. All data are presented as the mean ± SD (*n* = 4). The statistical analysis was performed using a two-tailed paired Student’s *t*-test (*p < 0.05, **p < 0.01, ***p < 0.001).

**Fig. 7:**
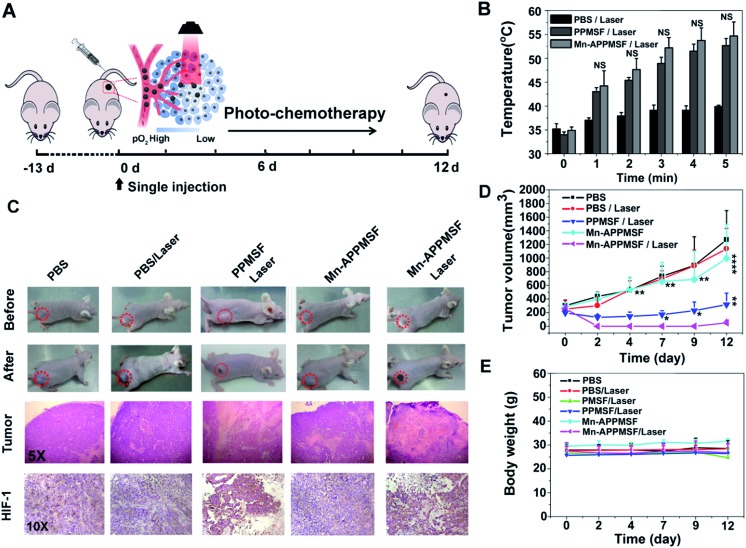
(A) *In vivo* response to PBS with or without laser irradiation, PMSF with laser irradiation, PPMSF with laser irradiation, and Mn-APPMSF with or without laser irradiation after treatment. (B) Temperature change of tumor sites undergoing laser irradiation (670 nm) under the indicated conditions (*n* = 4). The statistical analysis was performed using two-tailed paired Student’s *t*-tests (ns > 0.05). (C) Representative images and H&E results from tumors after 48 h of indicated treatment (5× magnification). *Ex vivo* immunohistochemical staining of tumor slices obtained from differently treated mice with/without laser irradiation (670 nm, 1 W cm^–2^, 5 min) after 24 h (10× magnification). (D) Tumor volume change of mice after indicated treatments. All data are shown as the mean ± SD (*n* = 4). The statistical analysis was performed using two-tailed paired Student’s *t*-tests (*p < 0.05, **p < 0.01, ****p < 0.0001). (E) Mean body weight change of mice in different groups after indicated treatments (*n* = 4).

Next, we evaluated the photothermal therapeutic efficacy of PPMSF *in vivo*. An IR thermal camera was employed to image the HepG2 tumor bearing nude mice after injection of PBS, PMSF and PPMSF, when exposed to 670 nm lasers (power intensity of 1W cm^–2^) for 5 min (Fig. 6A). The corresponding temperature change of tumors after various irradiation times is shown in Fig. 6B. As expected, the tumor temperature of PPMSF intravenously injected mice significantly increased from 39.9 ± 0.3 °C to 52.7 ± 1.5 °C within 5 min, and the temperature rise was much faster than that for PMSF injected mice (35.7 ± 0.4 °C to 44.5 ± 1.4 °C) at all time points due to the enhancement of DA in PPMSF. Meanwhile, the tumor temperature of PBS injected mice exhibited only a certain increase up to 39.2 ± 0.2 °C. It was found in our previous study that hyperthermia (*T* > 46 °C) induced by PTT could aggravate the hypoxia level in TMEs (unpublished). To confirm this phenomenon, immunohistochemical staining of hypoxia inducible factor (HIF-1) after indicated treatments (mentioned in the experimental section) was performed. As shown in Fig. 6C, compared with PBS treated mice with/without NIR laser irradiation (670 nm), the hypoxic areas were much more serious in PPMSF treated mice under 670 nm laser irradiation with a temperature of 52.7 ± 1.5 °C, while the hypoxic areas were not serious and similar to the control group in PMSF treated mice under the same laser irradiation with a temperature of 44.2 ± 1.4 °C. These results suggest that the high degree of hyperthermia induced by PTT could aggravate the hypoxia level in TMEs. Furthermore, we monitored and measured the tumor volumes using a Vernier caliper continuously for 12 days. As shown in Fig. 6D, the PBS treated mice with or without laser irradiation had fast tumor growth, suggesting that laser irradiation alone could not affect tumor growth. The mice treated with PMSF with laser irradiation exhibited a certain inhibition of tumor growth, but this still could not completely restrain the growth of tumors. In contrast, the mice treated with PPMSF exhibited more obvious inhibition of tumor growth and better therapeutic efficiency due to the high degree of hyperthermia. However, this still could not completely inhibit tumor growth and recurrence, which might be due to the existing resistance of tumor cells in the tumor hypoxia region as a consequence of PTT ablation. Thus, we assumed that the combination of PTT and the hypoxia-activated AQ4N prodrug might be an efficient strategy to enhance cancer therapy. As a proof-of-concept, we carefully studied the synergistic antitumor efficacy of Mn-APPMSF under laser irradiation (670 nm) in HepG2-tumor bearing mice. As shown in Fig. 7A and B, the tumor temperature of Mn-APPMSF intravenously injected mice significantly increased from 34.9 ± 0.8 °C to 54.6 ± 2.9 °C within 5 min, and the temperature rise was similar to PPMSF, leading to significant cell destruction and large amounts of damaged areas compared with other groups after 48 h of laser irradiation (Fig. 7C). The tumor tissues after 48 h of treatment were further evaluated by H&E staining. As shown in Fig. 7C, in PBS groups with or without laser irradiation, no obvious apoptosis or necrosis were identified in tumor slices, and the tumor cells kept their own normal morphology with clearly distinguishable membrane and nuclear structures. In contrast, Mn-APPMSF and PPMSF treated mice with NIR laser irradiation exhibited efficient cell destruction areas, indicating significant antitumor effects. To further compare the antitumor effects between Mn-APPMSF and PPMSF treated mice, we measured the tumor volumes with continuous monitoring for 12 days. As shown in Fig. 7D, Mn-APPMSF treated mice exhibited complete elimination of tumors after 2 days of being treated by laser irradiation (670 nm), and effectively inhibited tumor growth compared with the mice treated with PPMSF alone, which might cause by the synergistic therapeutic effects of PTT and PTT-induced hyperthermia aggravated serious hypoxia to enhance the AQ4N reduction. These results were further confirmed by HIF-1 immunohistochemical staining. As shown in Fig. 7C, the hypoxic areas were much more serious in Mn-APPMSF treated mice under laser irradiation (670 nm), and the results were similar to PPMSF treated mice.

To further investigate the PTT-enhanced hypoxia induced AQ4N activation at the tumor site, the metabolite of AQ4N (refer to as AQ4) was detected using HPLC analysis (the detailed experiments are available in the experimental section). As shown in Fig. S13,† the average concentration of AQ4N metabolite (AQ4) in the tumors with laser irradiation (0.701 μg g^–1^ on average, determined by HPLC) was significantly higher than that in tumors without laser irradiation (0.24 μg g^–1^ on average, determined by HPLC, p* < 0.05), demonstrating the PTT enhanced hypoxia induced AQ4N activation. The therapeutic dose of AQ4 after laser irradiation can be of benefit for combination therapy according to previous reports.^42^ Moreover, the AQ4N activation at tumor sites by hypoxia was further determined by electrospray ionization mass spectrometry (ESI-MS).^43^ As shown in Fig. S14,† the mass spectrum of the tumor sample after 670 nm laser irradiation displayed peaks at molecular weights of 445.14 and 413.05, corresponding to AQ4N [M + H]^+^ and the relevant AQ4 metabolite, respectively. These results demonstrate that AQ4N could be activated by hypoxic conditions. Importantly, the excellent therapeutic efficacy of Mn-APPMSF treatment did not lead to any weight loss, indicating the minimized side effects on mice (Fig. 7E). These results were subsequently verified by pathological examination of the major organs (including the heart, lung, spleen, liver, and kidney) by H&E staining at 12 days after indicated treatments (see the experimental section). As shown in Fig. S15,† there was no noticeable tissue damage in all the major organs, compared with the control group. The above results clearly suggest that the programmable combination of PTT and hypoxia-activated chemotherapy could be an efficient strategy to enhance cancer therapy with minimized side effects.

## Conclusions

We report a simple method that integrates SPs into a mesoporous silica framework and effectively loads the hypoxia-activated Fe(iii)-AQ4N prodrug through coordination effects for pH/hypoxia sequentially triggered and MRI guided cancer photo-chemotherapy. Using Mn-APPMSF nanohybrids, various advantages could be applied to cancer treatment including: (i) reduced SP/drug dissociation in physiological conditions, (ii) tumor micro-environment pH-triggered drug release and (iii) reduced risk of tumor recurrence after PTT treatment which aggravates hypoxia conditions to induce tumor cell resistance. Indeed, our Mn-APPMSF exhibited superior biocompatibility and excellent synergistic antitumor efficiency both *in vitro* and *in vivo*. These ideal antitumor effects could overcome the drawbacks of traditional PTT, which might offer a promising synergistic therapeutic strategy against cancers.

## Live subject statement

All animal experiments were performed strictly under the guidelines of the “National animal management regulations of China” and approved by the Animal Ethics Committee of Fujian Medical University.

## Conflicts of interest

The authors declare no competing financial interest.

## Supplementary Material

Supplementary information
