# Supplementary material for: pH/hypoxia programmable triggered cancer photo-chemotherapy based on a semiconducting polymer dot hybridized mesoporous silica framework
Source: Chem Sci. 2018 Jul 27;9(37):7390–9. doi: 10.1039/c8sc02408a (PMC6237124; doi:10.1039/c8sc02408a)
Supplement: Supplementary file 1 [file SC-009-C8SC02408A-s001.pdf]

Electronic Supplementary Material (ESI) for Chemical Science.
This journal is © The Royal Society of Chemistry 2018

Supporting information

pH / Hypoxia Programmable Triggered Cancer Photo-Chemotherapy Based on Semiconducting Polymer dot hybridized Mesoporous Silica Framework

Da Zhang[a, b], Zhixiong Cai[b,c], Naishun Liao[b], Shanyou Lan[b], Ming Wu[b], Haiyan Sun[d], Zuwu Wei[b], Juan Li[a]*, Xiaolong Liu[b]*

[a]MOE Key Laboratory for Analytical Science of Food Safety and Biology, State Key Laboratory of Photocatalysis on Energy and Environment, College of Chemistry, Fuzhou. University, Fuzhou 350116, P. R. China

[b]The United Innovation of Mengchao Hepatobiliary Technology Key Laboratory of Fujian Province, Mengchao Hepatobiliary Hospital of Fujian Medical University, Fuzhou 350025, P. R. China

[c]Key Laboratory of Biomedical Information Engineering of Ministry of Education, Institute of Biomedical Analytical Technology and Instrumentation, School of Life Science and Technology, Xi'an Jiaotong University, Xi'an 710049, P. R. China

[d]Department of Anesthesiology, Beijing Anzhen Hospital, Capital Medical University, Beijing 100029, P.R. China

*Corresponding Author:

lijuan@fzu.edu.cn; ORCID : 0000-0001-7175-2320

xiaoloong.liu@gmail.com; ORCID : 0000-0002-3096-4981

Materials.

Banoxantrone (AQ4N), dopamine-HCl, tetraethylorthosilicate (TEOS), cetyltrimethyl ammonium chloride (CTAC) and poly[2,6-(4,4-bis (2-ethylhexyl)-4H- cyclopenta-[2,1-b;3,4-b'] dithiophene)-alt-4,7-(2,1,3-benzothiadi-azole)] (PCPDTBT) were purchased from Sigma-Aldrich. PEG-$(NH_2)_2$ (Mw 2000) was purchased from PegBio. DI water (resistivity of 18.2 M Ω•cm) was obtained from the Milli-Q Gradient System (Millipore, Bedford, MA, U.S.), and was used for all studies. Unless clarified, all other chemicals were obtained commercially and applied as received.

Cell Culture. Hepatocellular carcinoma cell line HepG2 cells were obtained from ATCC (Manassas, VA). The cells were cultured in the RPMI 1640 medium which was supplemented with FBS (10%) and penicillin-streptomycin (100 IU/mL, Cellgro, Manassas, VA).

Preparation of PMSF nanoparticle. Firstly, 1 mL of tetrahydrofuran (TEA) mixture containing PCPDTBT (0.25 mg) was carefully injected into 10 mL DI water rapidly, then followed by sonication for 1.5 min using a microtip probe sonicator (40 W output) to prepare SPs[19]. Afterwards, the mixture was stirred at room temperature in dark overnight (600 rpm/min) to evaporate TEA. Subsequently, CTAC (2g) and triethanolamine (20 mg) were carefully dissolved in 20 mL DI-water (at room temperature), and the prepared SPs were then added into above mixture with vigor stirring for 1hr. Afterwards, the reaction mixture was heated to 80°C and then further stirred for 1hr; then, tetraethylorthosilicate (TEOS, 1.5 mL) was slowly and carefully added into mixture with stirring. After 2 hours of incubation under

stirring, the color of reaction mixture changed from ivory to wathet / blue with the formation of precipitates. Subsequently, the precipitates were collected, and then washed with 50 mL methanol (with 1 wt% NaCl) for three times to remove CTAC[24]. The final products of PMSF were then stored in ethyl alcohol for further usage.

Preparation of Mn-APPMSF. PPMSF was prepared by improving our previous method[27]. Briefly, 1 mg PMSF was added into 50 mL Tris-HCl solutions (10 mM, pH 8.5), then incubated for 2hrs. After centrifugation and washing with DI water and ethyl alcohol for twice, the obtained PPMSF was then mixed with PEG-$(NH_2)_2$ and $FeCl_3$ solution at pH 7.4 for 2hrs, and then 0.5 mg/mL of AQ4N was added. After incubation for 24hrs, the obtained APPMSF was further washed twice with DI water and obtained by centrifugation for 15 min. After that, APPMSF was mixed with $MnCl_2$ solution overnight. Subsequently, the obtained Mn-APPMSF was centrifuged for 15 min, then washed 2 times with DI water. Then, the final products were dispersed in PBS buffer (1mL, pH 7.4) for further usage. The amounts of Mn(II) and Fe(III) bound to Mn-APPMSF were further determined by ICP-MS) (Thermo, USA). Quantification of AQ4N was then measured by absorbance of supernatant at 600 nm. The standard curve with excellent linear correlation from 1-50 μg/mL was obtained (Y = 0.0038x-0.0029, R^2 = 0.999). Thus, the AQ4N in Mn-APPMSF (1 mg/mL) were determined to be 96 μg/mL, and the loading efficiency was 8.76%.

The photothermal conversion efficiency, η, was calculated using Equation 1 described by our previous reports[32], where h is the heat transfer coefficient, A is the surface area of the container, T_{max} is the equilibrium temperature, T_{Surr} is ambient temperature of the

surroundings, ΔT_{max} =T_{max}-T_{Surr}, I is incident laser power (1 W cm^{-2}), and A_λ is the absorbance of PMSF or PPMSF at 670 nm. Q_s is the heat associated with the light absorbance of the solvent, which is measured independently to be 25.2 mW by using deionized water without nanoparticles as the solvent.

$$\eta = \frac{hA\Delta T_{max} - Q_s}{I(1-10^{-A_\lambda})} \qquad (1)$$

The value of hA is derived according to Equation 2:

$$\tau_s = \frac{m_D C_D}{hA} \qquad (2)$$

Where τ_s is the sample system time constant, m_D and C_D are the mass (1 g) and heat capacity (4.2 J g^{-1}) of deionized water used as the solvent, respectively.

In order to obtain the hA, herein introduce θ, which is defined as the ratio of ΔT to ΔT_{max}:

$$\theta = \frac{\Delta T}{\Delta T_{max}} \qquad (3)$$

hA can be determined by applying the linear time data from the cooling period versus -lnθ (Figure S5). Substituting hA value into Equation 1, the photothermal conversion efficiency (η) of PMSF and PPMSF can be calculated.

For the PMSF:

$hA = m_D C_D/\tau_s$ = 4.2 J/245.10; $A\lambda$ = 0.1025; τ_s = 245.10; I = 1 W cm^{-2}; ΔT_{max} = 5.0 °C

η = ((4.2/245.10)×5.0-0.0252)/(1×(1-10$^{-0.1025}$)) = 0.060479/0.21023=28.77%

For the PPMSF:

$hA = m_D C_D/\tau_s$ = 4.2 J/257.54; $A\lambda$ = 0.5014; τ_s = 257.54; I = 1 W cm^{-2}; ΔT_{max} = 24.3°C

η = ((4.2/257.54)×24.3-0.0252)/(1×(1-10$^{-0.5014}$)) = 0.371088/0.684790=54.19%

pH sensitive drug release of Mn-APPMSF. The pH responsive release study was investigated as follows: Mn-APPMSF (0.5 mg, PPMSF; 48 μg/mL, AQ4N) was dissolved

into PBS buffers (1 mL) with various pH values (pH 5.0 and 7.4); at certain time intervals, 0.5 mL supernatant was carefully taken and analyzed to calculate the amounts of released drug by measuring the supernatant absorbance at 600 nm after the centrifugation at 13000 rpm/min for 10 min. To keep the constant volume, 0.5 mL PBS with corresponding pH values was further added after each sampling.

Confocal microscopy and flow cytometry studies. The cell uptake of Mn-APPMSF by HepG2 cells was analyzed by using a confocal laser scanning microscope. In the typical experiment setting, the HepG2 cells (5×10^4) were seeding in 35-mm glass-bottom Petri dishes and cultured for another 24hrs at 37˚C in the incubator; afterwards, the Mn-APPMSF were added into the cell culture dish and further incubated for 0.5, 1, 1.5, 2 and 4hrs, respectively. Then, the treated cells were further washed for 3 times with PBS buffer (pH 7.4) and then fixed with 4% paraformaldehyde buffer for 15 min. Afterwards, 4',6- diamidino-2-phenylindole (DAPI) was carefully added into cell culture dishes, and incubated for 15 min in dark at room temperature. Finally, the stained cells were analyzed by the confocal microscope (Carl Zeiss LSM 780, Germany), with 405 nm laser excitation for DAPI and 633 nm laser excitation for AQ4N.

To conduct the flow cytometry analysis, the HepG2 cells were carefully seeding to 6-well plates with the cell density of 5×10^5 cells/per well, and then the cells were incubated in incubator with 5% CO_2 and the humidity atmosphere for 24hrs. Afterwards, the original cell culture medium was carefully replaced by the fresh cell culture medium that contains Mn-APPMSF (0.02 mg/mL, PPMSF; 1.92 μg/mL, AQ4N); after further incubation for another

4hrs, the cells then were washed 3 times with PBS buffer and then irradiated by NIR laser (670 nm, power intensity of 1W/cm^2) for 5 min. After that, the cells was collected and dispersed in 1 mL PBS buffer. After that, cells were carefully filtered by the nylon mesh (40 microns) to remove cell aggregates before FACS analysis. The fluorescence measurement of intracellular AQ4N was conducted in FL4 channel (excitation at the 670 nm).

Antitumor efficacy of Mn-APPMSF *in vitro.* CCK8 (Cell Counting Kit) assay was applied to investigate antitumor effects of Mn-APPMSF against HepG2 cells. Briefly, cells were carefully seeded in the 96-well plate with the density of 1 × 10^4 cells/per well, and then incubated with 5% CO_2 in an incubator with humidity atmosphere for 24hrs. Afterwards, the original medium was discarded; subsequently the cells were further washed 3 times with PBS buffer to carefully remove dead cells, then followed by the incubation with PMSF, PPMSF and Mn-APPMSF for 4hrs, and subsequently washed 2 times with PBS to carefully remove non-internalized nanoparticles. Then the 96-well plate was irradiated with NIR laser (670 nm, power intensity of 1 W/cm^2) under aerobic or hypoxia condition, respectively. The cells only incubated with the culture medium were taken as untreated control. Cell viability was calculated as follows: Cell viability = (ODsample - ODblank) / (ODcontrol - ODblank). The ODsample and ODcontrol were absorbance values of treated cells (as indicated) and untreated cells, respectively. ODblank was the absorbance values of the CCK8 reagent at 450 nm.

Antitumor efficiency of Mn-APPMSF *in vivo.* The male BALB/c-nude mice (about 5 weeks old) were obtained from China Wushi, Inc. (Shanghai, China). All animal experiments

were performed strictly under the guidelines of the "National animal management regulations of China" and approved by the Animal Ethics Committee of Fujian Medical University. The mice with tumor-bearing were established through subcutaneously injection of HepG2 cell suspension (5×10^6 cells) in the sterilized PBS. If the tumor size has reached around ~5 to 7 mm, 150 μL Mn-APPMSF (1 mg/mL, PPMSF; 96 μg/mL, AQ4N) were intravenously injected into each mouse. One groups of mice treated with the same volume of sterilized PBS was taken as the control. The mice were then segregated into following five groups:

(i) sterilized PBS without the laser irradiation (n = 4);

(ii) sterilized PBS with the laser irradiation of 5 min (n = 4);

(iii) PMSF with the laser irradiation of 5 min (n = 4);

(iv) PPMSF with the laser irradiation of 5 min (n = 4);

(vi) Mn-APPMSF without laser irradiation (n = 4);

(vii) Mn-APPMSF with the laser irradiation (1 W/cm^2) of 5 min (n = 4).

For antitumor efficiency analysis, the irradiation was carefully conducted after 4hrs of injection. Then, the therapeutic efficiency was carefully evaluated by monitoring the tumor volume change and body weight change in each group every 2 days, and over 12 days in total. The tumor size was carefully measured by the caliper every other day after indicated treatment. Finally, tumor volume (V) was calculated by following equation:

$$V = A*B^2 / 2$$

Here, the A is longer diameter and B is the shorter diameter (mm) of tumor, respectively.

We further investigated the PTT-enhanced hypoxia induced AQ4N activation at tumor site by detecting the metabolite of AQ4N (refer to as AQ4) through ESI-MS and HPLC analysis. The experiments were performed as follows: After intravenous injection of Mn-APPMSF for 4hrs, the mice (n=3) was irradiated by 670 nm laser at the tumor site with the power intensity of (1 W/cm^2) for 5 min. The mice injected with Mn-APPMSF but without laser irradiation was taken as control. After 24hrs of irradiation, the tumors were excised, weighted and then crushed by sonifier in MeCN solution (containing 0.2% H_2O_2) [10, 42, 43]. Afterwards, the tumor samples were homogenized using a pellet pestle, and centrifuged at 10000g for 5 min. Then, the supernatant was dried under vacuum evaporation, and afterwards reconstituted with DMSO. Subsequently, 20 μL of reconstituted supernatant was injected into the HPLC (Agilent 1260 Infinity, Agilent Technologies, Germany), and analyzed using Agilentzorbax Eclipes Plus C18 colum (4.6 mm x 100 mm, 3.5 um, Agilent Technologies, USA) by isocratic elution with 78% of 50 mM ammonium formate (pH 3.6) and 22% of acetonitrile. The column temperature was maintained at 45°C, and the flow-rate was maintained at 0.6 mL/min. Standards of AQ4 were prepared and diluted in DMSO. The AQ4 concentration was monitored by the absorption at 276 nm. The correlation between the peak area at the retention time of 3.27 min and the concentration of AQ4 was analyzed by linear regression (Y = 160.05x - 5.7083), which showed a well-correlated linear relationship (R^2 = 0.9981); meanwhile, the calibration curve was prepared from 0.05 to 10 μg/mL. Moreover, the AQ4N activation at tumor site by hypoxia was further determined by electrospray lionization mass spectrometry (ESI-MS)[43].

Histological evaluation and long-term toxicity examination. To further carefully evaluate histological changes of the tumors after indicated treatment, a tumor-bearing mouse in each group were then sacrificed after 48hrs of treatment; afterwards, the tumors were collected, sectioned and then stained with Hematoxylin and eosin (H&E) for histopathology analysis. Immunohistochemical staining of the tumor slices was performed by antibodies specific for HIF-1 (1:800) (H1alpha67, Novus Biologicals, Littleton, CO or 54, BD Bioscience) after indicated treatment for 24hrs. The long-term toxicity examination of mice was performed as follows: one tumor-bearing mouse in each group were sacrificed at the day 12 after indicated treatment, and major organs (including heart, liver, spleen, lungs and kidney) of the mouse were collected, then fixed in the 4% neutral formaldehyde, embedded in the paraffin, stained with the H&E, and finally observed under the Zeiss microscope (Axio Lab.A1).

Statistical analysis: Statistical analysis of data was performed using one-way of variance (ANOVA) method or the two-tailed paired Student's T-test, *$p<0.05$, **$p<0.01$, ***$p<0.001$. All the data were shown as means ± SD through at least three experiments.

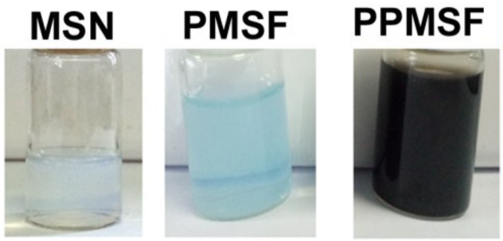

Fig. S1. The photographs of MSN, PMSF and PPMSF in DI-water.

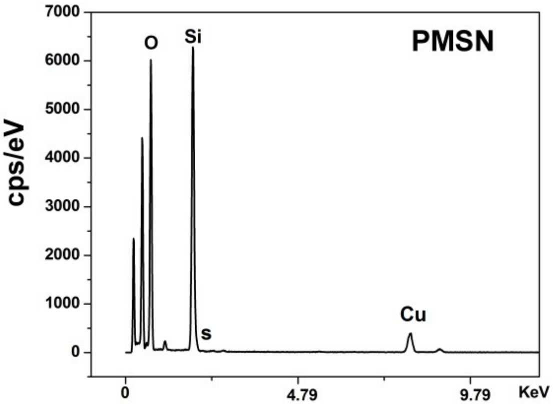

Fig. S2. Energy dispersive x-ray spectroscopy of PPMSF for the element of O, Si, and S.

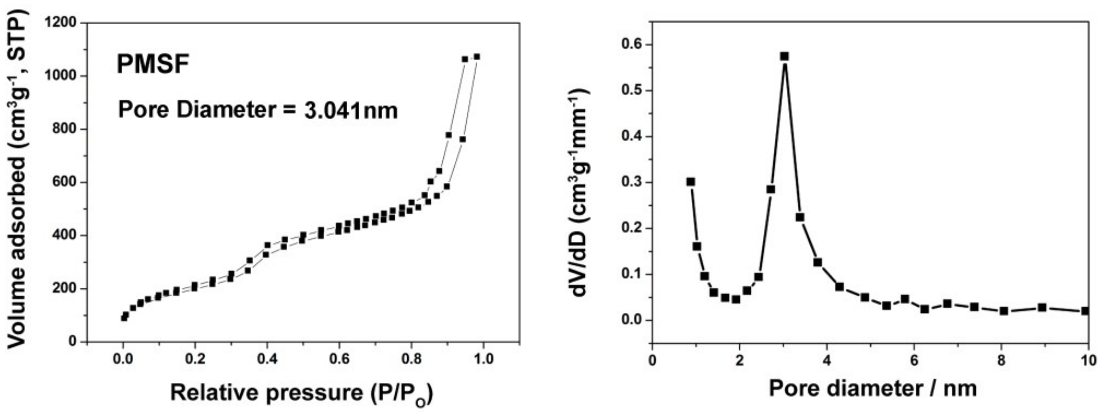

Fig. S3. N_2 sorption isotherm and DFT porous size distribution for PMSF.

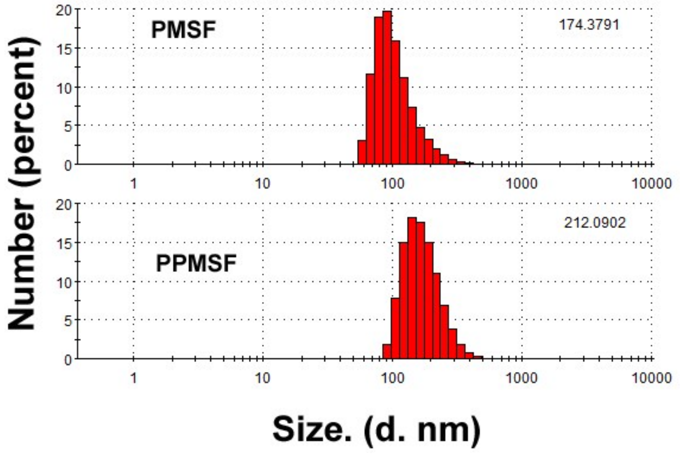

Fig. S4. The size distribution of PMSF and PPMSF in PBS buffer determined by DLS.

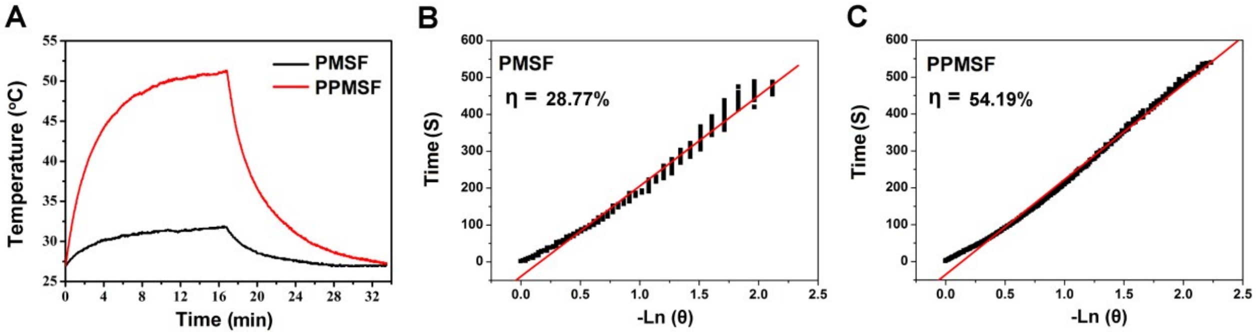

Fig. S5. (A) The photo-thermal conversion ability of PMSF and PPMSF with NIR laser irradiation for 1000 s (670 nm, 1 W/cm^{-2}), and then the laser was shut off. (B) Linear fitting of the time data obtained from the cooling period versus -*lnθ*.

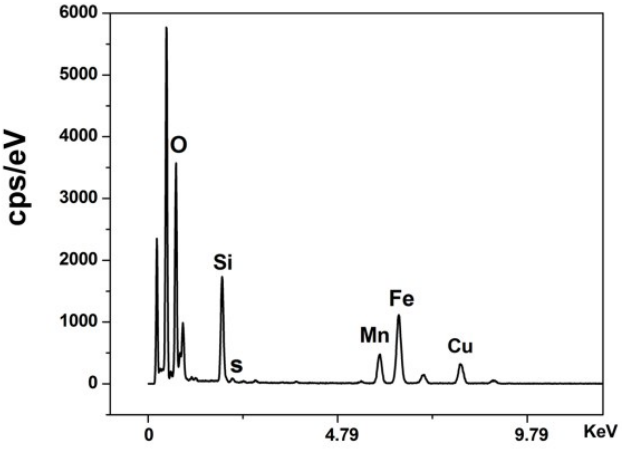

Fig. S6. Energy dispersive x-ray spectroscopy of Mn-APPMSF for the element of O, Si, S, Fe and Mn.

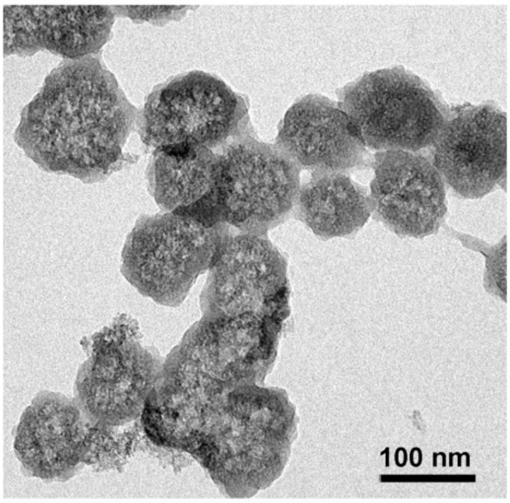

Fig. S7. Representative TEM image of Mn-APPMSF.

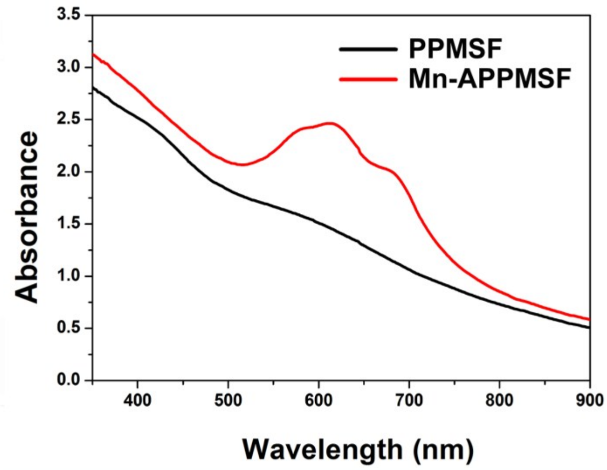

Fig. S8. UV-vis-NIR spectra of PPMSF and Mn-APPMSF.

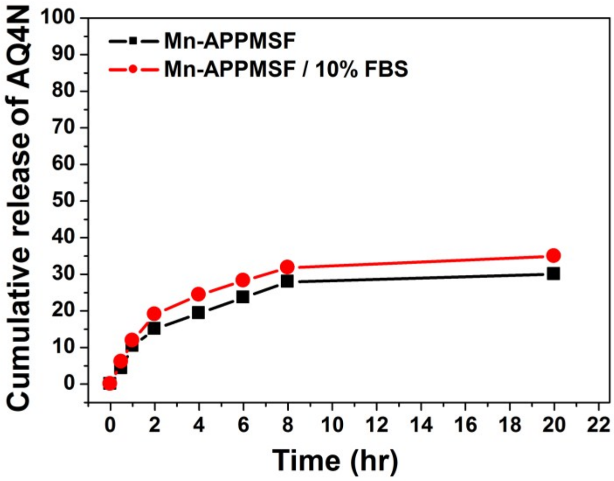

Fig. S9. *In vitro* AQ4N release kinetics from Mn-APPMSF in PBS buffer (pH 7.4) with or without 10% FBS at 37°C.

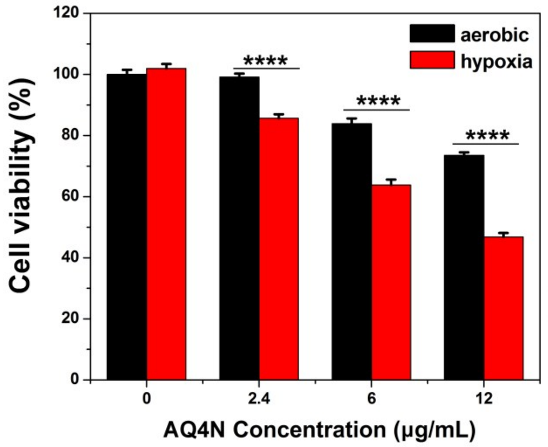

Fig. S10. Cell viability of HepG2 cells treated with free AQ4N for 48hrs in aerobic and hypoxia condition (1% O_2) (n = 5).

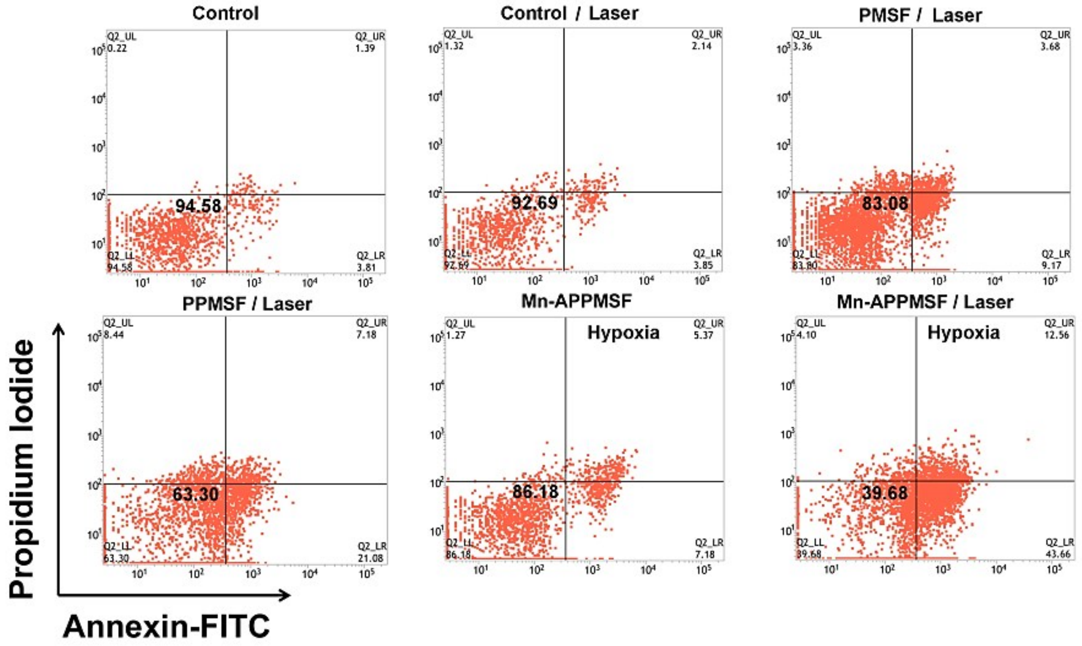

Fig. S11. Apoptosis of HepG2 cells incubated with PBS, PBS + laser, PMSF + laser, PPMSF + laser, Mn-APPMSF in hypoxia, Mn-APPMSF + Laser in hypoxia. The cells apoptosis were determined by flow cytometry analysis using Annexin V-FITC/ PI staining. The laser power intensity at 670 nm was 1 W/cm^2 for 5 min.

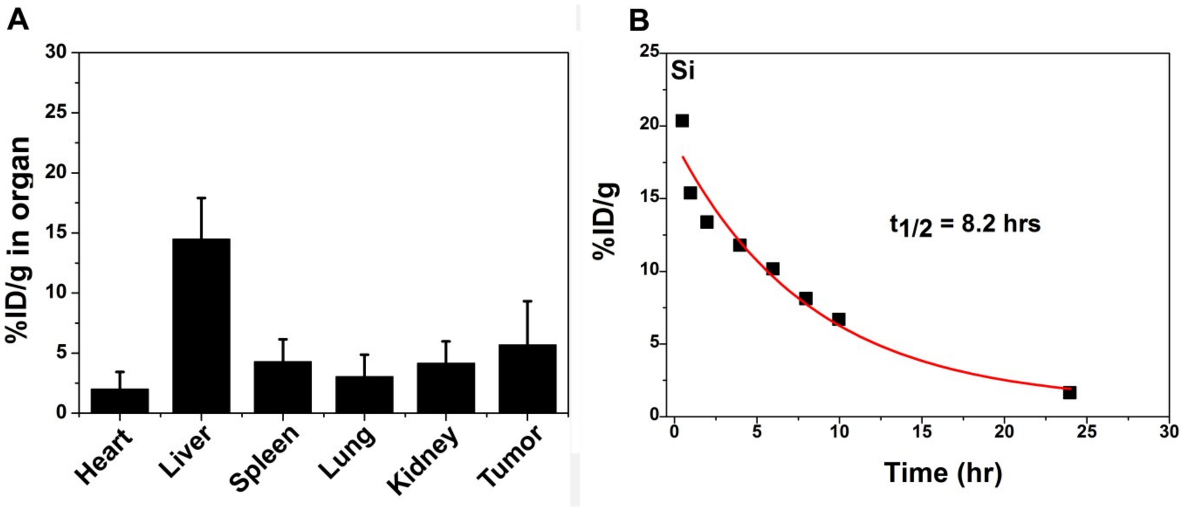

Fig. S12. (A) Organ and tumor distribution of Mn-APPMSF after intravenous injection into HepG2 tumor bearing mice for 4hrs (n = 3), which was determined by the ICP-MS analysis of Si (only existed in our nanocomposite). (B) *In vivo* pharmacokinetics of Mn-APPMSF in blood after intravenously injecting our Mn-APPMSF into tumor-bearing mice.

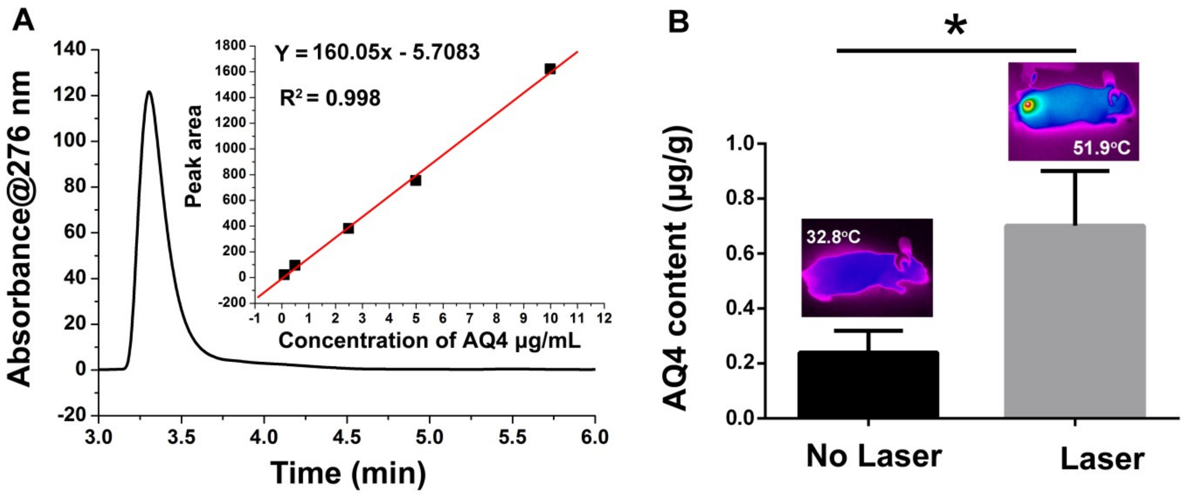

Fig. S13. (A) Chromatogram of the AQ4 standard (10 μg/mL), and the peak area of various concentration of AQ4 from 0.05 μg/mL to 10 μg/mL at the retention time of 3.27 min. (B) The content of metabolite AQ4 was detected by HPLC in tumor samples of Mn-APPMSF

injected mice with or without laser irradiation, respectively. The statistical analysis was performed with the two-tailed paired Student's T-test, $*p<0.05$ (670 nm, 1 W/cm^2).

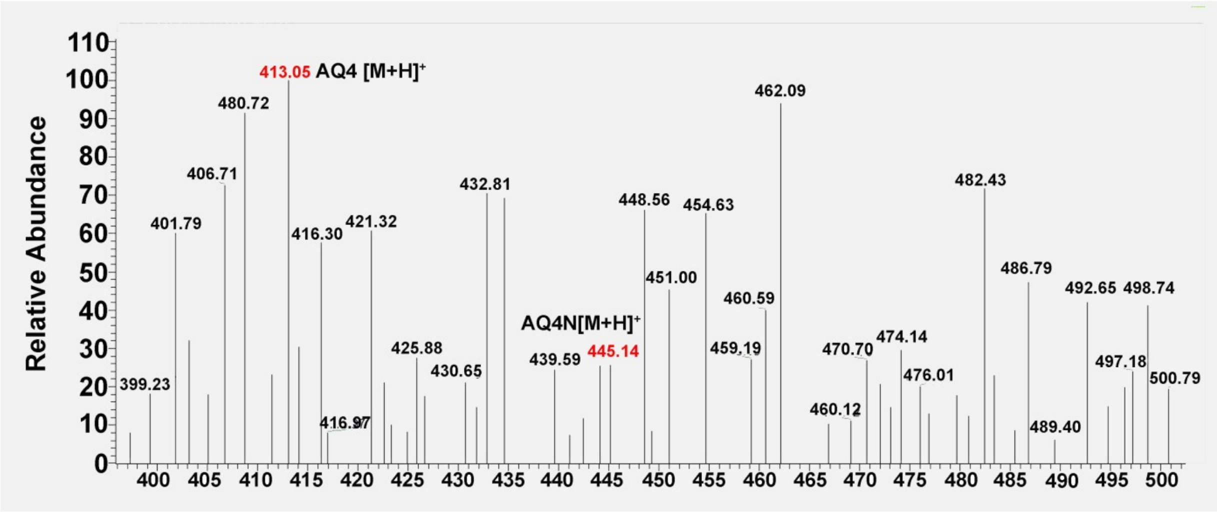

Fig. S14. MALDI mass spectra of AQ4N and AQ4 in tumor tissue after 670 nm laser irradiation (670 nm, 1 W/cm^2).

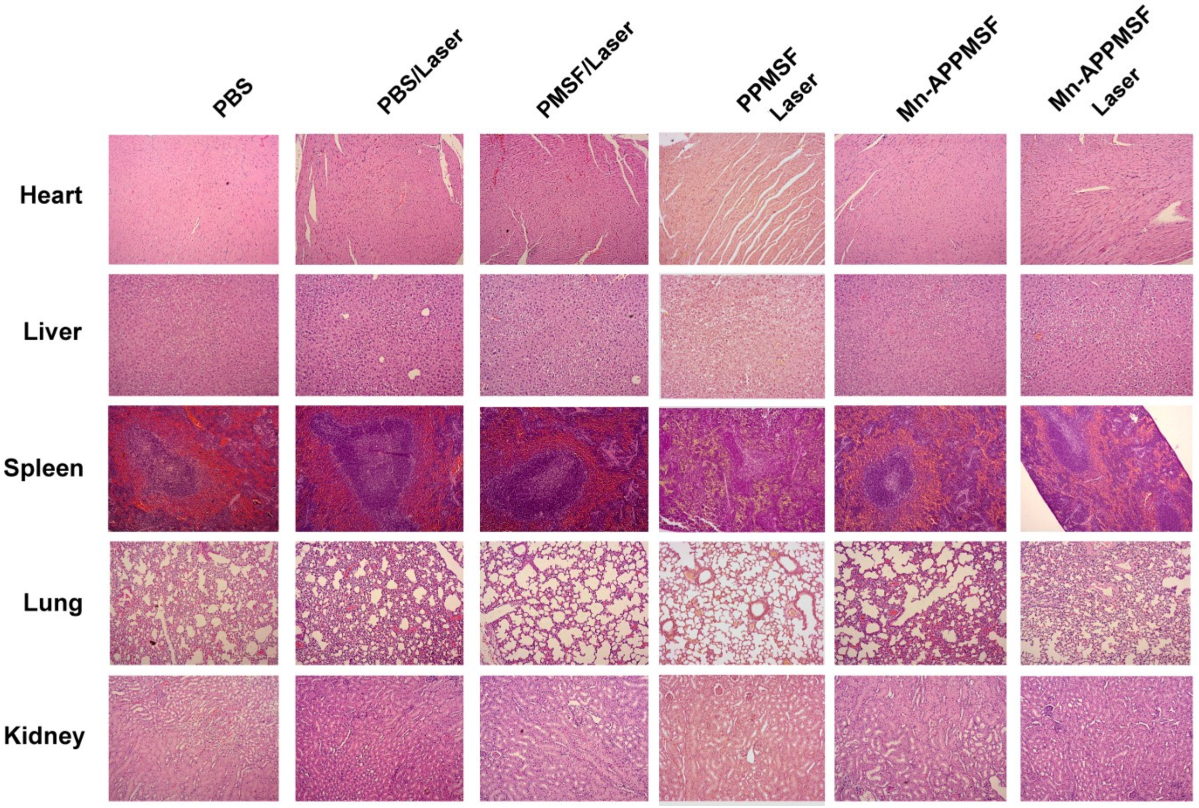

Fig. S15. The pathological changes of main organs evaluated by H&E staining which were acquired at 12 days after different treatment. No noticeable pathological changes were observed in these organs.
